# Bioactivity of a Novel Glycolipid Produced by a Halophilic *Buttiauxella* sp. and Improving Submerged Fermentation Using a Response Surface Method

**DOI:** 10.3390/molecules21101256

**Published:** 2016-09-22

**Authors:** Abdolrazagh Marzban, Gholamhossein Ebrahimipour, Abolghasem Danesh

**Affiliations:** 1 Biotechnology Research Center, Mashhad University of Medical Sciences, Mashhad 91775-1365, Iran; marzban86@gmail.com; 2 Department of Microbiology, Faculty of Biological Sciences, Shahid Beheshti University, Tehran 19839-4716, Iran; G-ebrahimi@sbu.ac.ir

**Keywords:** glycolipid biosurfactant, antimicrobial activity, *Buttiauxella*, response surface method

## Abstract

An antimicrobial glycolipid biosurfactant (GBS), extracted and identified from a marine bacterium, was studied to inhibit pathogenic microorganisms. Production of the GBS was optimized using a statistical method, a response surface method (RSM) with a central composite design (CCD) for obtaining maximum yields on a cost-effective substrate, molasses. The GBS-producing bacterium was identified as *Buttiauxella* Species in terms of biochemical and molecular characteristics. This compound showed a desirable antimicrobial activity against some pathogens such as *E. coli*, *Bacillus subtilis*, *Bacillus cereus*, *Candida albicans*, *Aspergilus niger*, *Salmonella enterica*. The rheological studies described the stability of the GBS at high values in a range of pH (7–8), temperature (20–60) and salinity (0%–3%). The statistical optimization of GBS fermentation was found to be pH 7, temperature 33 °C, Peptone 1%, NaCl 1% and molasses 1%. The potency of the GBS as an effective antimicrobial agent provides evidence for its use against food and human pathogens. Moreover, favorable production of the GBS in the presence of molasses as a cheap substrate and the feasibility of pilot scale fermentation using an RSM method could expand its uses in food, pharmaceutical products and oil industries.

## 1. Introduction

Biosurfactants, which are synthesized by a variety of living organisms, are amphipathic molecules that have numerous applications in the food, oil, cosmetic and medical industries [1]. In recent years, they have drawn attention from many researchers for use as emulsifying agents in the food processing, oil recovery and pharmaceutical industries [2,3,4]. GBSs are classified into four categories: (i) Rhamnolipids produced by *pseudomonas* species [5]; (ii) Trehalose lipids released mainly from *rhodococci* [6]; (iii) Xylolipid synthesized by *lactobacilli* [7]; and (iv) Sophorolipids obtained from *candida* species [8].

Biosurfactants as a green alternative are preferred over synthetic surfactants owing to their lower toxicity, higher biodegradability and great stability at different physiochemical conditions [9]. The capability of biosurfactants to reduce surface tension and form stable emulsions is a virtue for countless applications [10]. Besides, biosurfactants have appeared to inhibit some pathogenic organisms. Several studies have suggested some potent biosurfactants with broad spectrum activity against human, plant and food pathogens [8,11,12]. They have attributed the antimicrobial and anti-adhesive activities to the amphipathic nature of biosurfactants, especially the hydrophobic tail. In line with raising awareness about emerging antibiotic resistance among pathogens, biosurfactants may have surmounted the cumbersome exercises to discover new antimicrobial agents [13].

The present research intends to elucidate the structure of an antimicrobial glycolipid isolated from a marine halotolerant bacterium identified phylogenitically as a *Buttiauxella* species. Optimization of glycolipid production was also conducted by response surface method (RSM) in terms of antimicrobial activity of crude antimicrobial glycolipids under different cultural conditions. In addition, the surface active property, oil spreading efficiency and the emulsification activity of the GBS have been examined.

## 2. Results

### 2.1. Bacterial Characterization and Antimicrobial Studies

The isolated bacterium was identified as a halotolerant *Gamma Proteobacterium* belonging to the *Enterobacteriales* family. Biochemical and morphological examinations revealed to be *bacillus* (rod-shaped), gram-negative, motile, catalase-positive and oxidase-negative. The *Buttiauxella* species is one of the members of enterobacteriaceae that could be found in various habitats. The strain had 99% similarity to *Enterobacter* sp. as concluded via DNA BlastN in NCBI Genbank. Accordingly, the isolate was identified as *Buttiexella* sp. M44 and the associated 16S rRNA sequence was deposited in Genbank with accession number KU350741.

The glycolipid BSs produced by *Buttiauxella* sp. M44 exhibited a different inhibitory effect on the test microorganisms (Table 1). Antimicrobial activity was highly potent against *C. albicans*, *A. niger* and then *E. coli*. A moderate potential was found to inhibit the growth of *S. enteric*, *B. subtilis*, *B. cereus* and *S. aureus* while no zone of inhibition was observed against *P. aeruginosa*.

### 2.2. Compositional Characterization of by GC-MS

**Figure 1:**
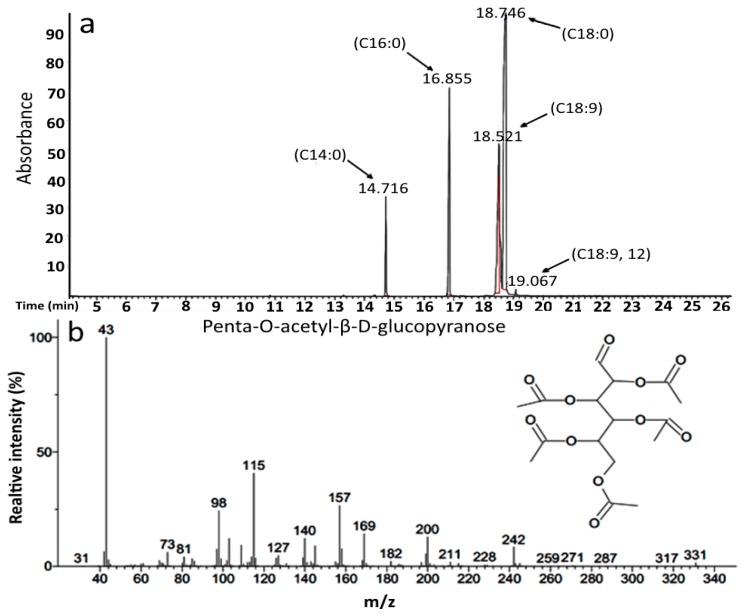
TIC profiles of gas chromatography (**a**) fatty acid portion showed at least 5 fatty acid derivatives and (**b**) glycosylic portion of glycolipid produced by *Buttiauxella* sp. M44.

GC-MS analysis shows that a major part of the antimicrobial glycolipid produced by the bacterium is probably related to a glucose-derived sugar linked to a fatty acid moiety such as octadecanoic acid and 9-octadecenoic acid, while the remaining compounds comprise four derivative analogues with the only difference in their fatty acid moieties (Table 2). The GC chromatogram and the corresponding mass spectrum for each compound are presented in Figure 1a,b.

### 2.3. Characterization of GBS by FT-IR

**Figure 2:**
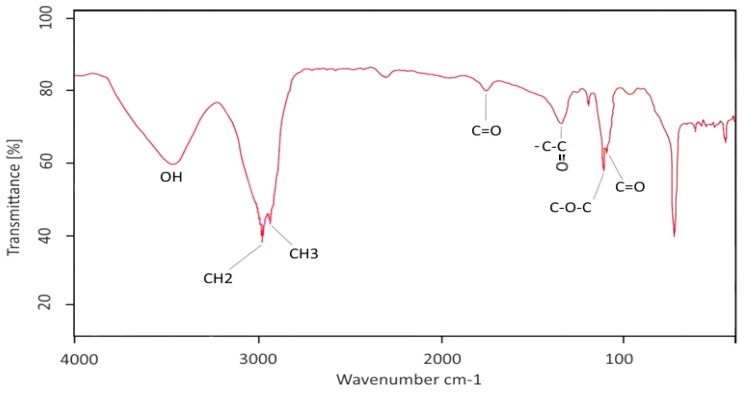
Fourier transform infrared (FTIR) spectrum of glycolipid biosurfactant.

FT-IR spectrum displays a broad peak at 3435.42 cm^−1^ elucidating OH group (Figure 2). Two stretch signals at 2940.29 and 2877.33 could be associated with CH_2_ and CH_3_ in the hydrocarbon chain. A lowered peak at 1769.34 cm^−1^ proves the presence of a carbonyl group (C=O) conjugated with sugar as well as a deformed signal at 1374.82 affected by the carboxyl group. Additionally, three stretch signals appeared at 1213.84, 1121.24 and 1118.04 cm^−1^ where the former indicates a C–O–C bond and the two latter confirm the presence of C=O in the glycolipid structure [14]. The region between 700 and 950 cm^−1^ is considered a carbohydrate fingerprint and often presents some peaks related to anomeric carbon [15].

Carbon and hydrogen NMR (^13^C- and ^1^H-NMR) were performed to elucidate the structure of the purified GBS. Table 3 describes the ^13^C- and ^1^H-NMR spectrums of GBS, d-glucopyranose and octadecanoic acid. The chemical shift of the signals related to sugar and lipid moiety as compared to corresponding peaks of d-glucose and octadecanoic acid denotes the molecular structure of GBS. The major peaks (as shown by star) indicate how fatty acids are linked to the sugar moiety. On comparison to ^13^C-NMR spectrum of octadecanoic acid, it can be confirmed that a marked shift away from about 181.7 ppm has occurred and the related peak has been transferred to 172.8 ppm, indicating that the carboxyl group in fatty acids are covalently attached to OH-C6 from the sugar moiety [16,17]. Moreover, lack of ^1^H-NMR peaks related to OH-C6 from glucopyranose and COOH from fatty acid indicates that an ester bond has been created between OH-C6 of the sugar and COOH of fatty acid in GBS.The ^1^H-NMR profile for GBS structure displays multiple peaks at 3.3 to 4 ppm, while lacking carboxylic group peak at 10–12 ppm, indicating lipid-to-sugar ester conjugation [17].

### 2.4. Effect of pH, Temperature, and Salinity on GBS Activity

The rheological parameters of GBS after treating for 1 h in the range of pH, temperature and salt have been presented in the Table 4. Crude GBS exhibited a considerable ability to reduce surface tension (ST) in the range of pH 7–8, temperature of 20–60 °C and salinity of 0%–3%. The maximum bioemusification activity (E24) was observed in the pH range of 7–8, which was about 47%. The effect of temperature was not significant on the E24 value produced by the bioactive compound from 20 to 100 °C. Overall, the increase in NaCl concentration decreased E24 produced by the bioactive compound. Oil spreading activity remained aroind 11 mm for all treatments and no change was observed in different conditions.

### 2.5. Optimization of Glycolipid Production by RSM

Antimicrobial activity was determined by plotting diameters of zones of inhibition against reciprocates of the dilution factors followed by calculating the area under the dilution curve (AUDC) as response. Based on the experimental data in which those insignificant terms removed using a stepwise regression and the output formula achieved from RSM with CCD, the following quadratic model was proposed.

Y = + 6.74 + 1.19X_1_ − 0.32X_2_ + 0.41X_1_X_2_ + 0.44X_1_X_5_ − 0.71X_2_X_4_ − 0.53X_3_X_5_ − 0.23X_1_^2^ − 1.40X_2_^2^ + 0.33X_4_^2^

Experimental data in 32 trials are shown in Table 5 as actual and predicted values. Table 6 represents the results of the ANOVA relative to the quadratic model. The model was significant, indicating the ability and accuracy of the model in predicting responses (*p* < 0.0001). In addition, an insignificant lack of fit (*p* >> 0.05) for the model indicated that the model fits the experimental data perfectly. The predicted R squared of 0.7476 agreed with the adjusted R squared demonstrating strong correlation between experimental and predicted values. Finally, adequate precision of 21.158, evaluating the signal-to-noise ratio, revealed a strong signal compared to noise. A value above 4 (signal-to-noise ratio > 4) denotes adequate precision of the model. The influence of the variables on antimicrobial activity as linear coefficients was found to be strongly significant for pH (X_1_) and temperature (X_2_). Interactive coefficients were significant for X_1_X_2_, X_1_X_5_, X_2_X_4_ and X_3_X_5_. The quadratic coefficients for the model were significant for X_1_^2^, X_2_^2^ and X_4_^2^. As seen in Table 6, coefficient of variation (C.V. = 7.91) suggests that the model is reliable and the response values are repeatable.

**Figure 3:**
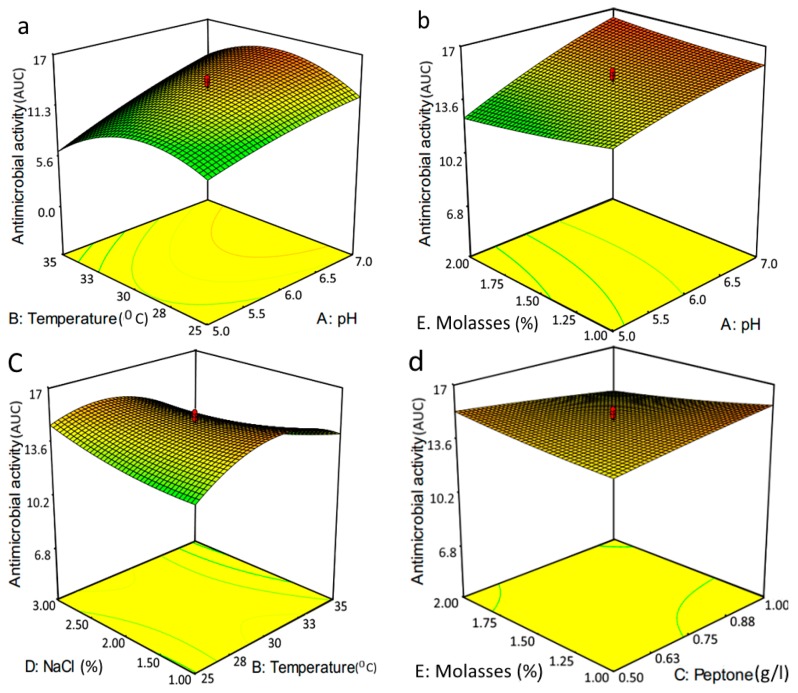
Three-dimensional surface plots. Five significant terms between variables: (**a**) interactive effects between temperature and pH exhibited a polynomial curvature; (**b**) molasses and pH have no curvature, but affected significantly polynomial; (**c**) NaCl and Temperature have significant polynomial curvature and (**d**) molasses and peptone have interactive polynomial effect, but no curvature.

The fitted three dimensional plots show the effects of the independent factors with significant interactions with the variable pairs (Figure 3). The shape of these plots could be helpful to understand the model and the status of interaction between the independent variables. The optimum predicted responses are shown at highlighted points based on interactive effects between each pair of independent factors. As per the model, the maximum predicted response for antimicrobial activity could be estimated at 17.26 at the desirable conditions, including pH 7, temperature 33 °C, peptone 1%, NaCl 1% and molasses 1% (*w*/*v*).

### 2.6. Time Course Study of Bacterial Growth and GBS Production

**Figure 4:**
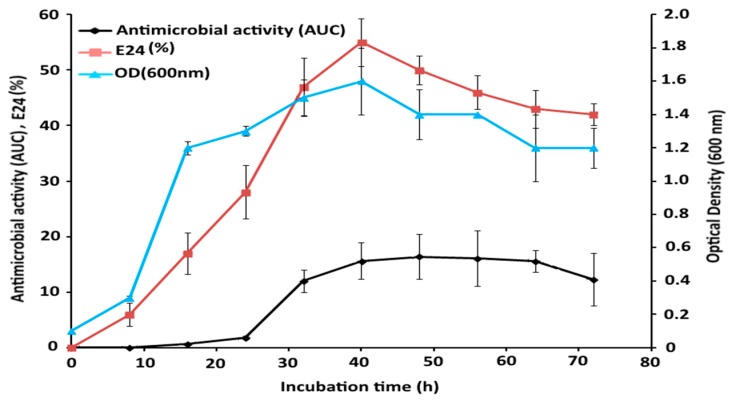
Time course for production of the glycolipid by the bacterium. Antimicrobial activity, bacterial growth and emulsification activity were determined during 72-h incubation at the optimized conditions suggested based on RSM design.

The experiment was conducted according to the predicted parameters of RSM for optimum antimicrobial activity. The antimicrobial and emulsification (E24) activities accord with bacterial growth (OD 600), which increased up to 42 h post-incubation (Figure 4). The antimicrobial activity remained constant during the following hours of the incubation, while emulsification activity decreased along with bacterial growth. Moreover, the antimicrobial activity reached around the predicted response, denoting the reliability of RSM prediction. The time course study shows a maximum inhibitory effect around 15.5 AUDC, meaning that it presents a significant correlation with the predicted value (AUDC 17.5) calculated by the RSM statistical algorithm. Besides, this difference between the RSM predicted and time course responses may be due to unknown and unpredictable conditions.

## 3. Discussion

*Buttiauxella* species is one of the members of enterobacteriaceae found in various habitats. Up to now, there have been a few reports on the antimicrobial activity of *Buttiauxella* species. Furthermore, the antimicrobial activity of other enterobacteriaceae has been reported in the literature. It has been reported that *Serratia marcescens* variants produced bioemulsifying compounds with antimicrobial activity [18]. Dusane et al. [19] described a glycolipid bioemulsifier with anti-biofilm property produced by a marine strain of *Serratia marcescens*. In the current investigation, it was found that the *Buttiauxella* (one of the nearest species to *Entrobacter* subspecies.) could produce a set of glycolipids with concomitant antimicrobial and bioesmulsifing activity, where the glycolipids consist of a glucose linked to varying C14, C16 and C18 fatty acid moieties [14]. To our knowledge, this is the first time that the molecular structure of the potential bioemulsifing compounds (GBSs) have been scrutinized with the methods discussed in the “Experimental” section. Given the variation in the carbon length of the molecules, it is plausible for the GBS to manifest antimicrobial activity against a broader spectrum of microorganisms with superior activity as compared to other GBSs [20]. It was, therefore, noteworthy that we achieved the optimum conditions for GBS production, given cultural factors that may affect the production [21].

The optimization of a fermentation process is a common action leading to low-cost production, especially when the substrate for the microorganism growth and productivity is economically cost-effective [22]. In this regard, statistical design methods could contribute to producing conclusive optimum conditions for the production. Response surface methods have been substantiated to have a practical advantage over conventional methods for optimizing a fermentation process [5]. In the current investigation, a molasses batch was utilized as an inexpensive energy and carbon source for bacterial growth and bioemulsifier production [23,24]. Given the optimum conditions achieved by the RSM, it was found that the bacteria could produce the maximum yield when the temperature was set at 33 °C, which is amenable for application in large-scale production without additional cooling equipment or consuming energy. Since bacterial growth and metabolism results in elevating the temperature of the incubating system, the mentioned temperature could be spontaneously achieved in the fermentation system [25,26]. Moreover, the AUDC of the antimicrobial activity in different dilutions was applied as a rough indicator of the production of the more relevant GBSs with high antimicrobial activity [27]. It seems plausible that the optimum conditions are also optimal for the production of the GBSs that exhibit superior antimicrobial activity as compared to other GBSs produced by the bacterium. Profiling the GBS production when the bacterium faces different cultural conditions would be an interesting issue that merits further investigation. The same study also merits consideration with respect to other species producing multiple emulsifiers with antimicrobial activity. On the other hand, the extracellular entity of the GBSs is regarded as an advantage in the purification steps of the GBSs from the bacteria with high feasibility in the food processing industry [28]. Substituting peptone, used as the nitrogen and protein source in the RSM study, with other less expensive alternative sources such as whey might further reduce the cost of the GBSs production [29]. The current GBSs, having different hydrophobic lengths, might also be suited for oil remediation, as it suggests that they might have different hydrophobic–lipophilic balances, suitable for emulsifying diverse hydrocarbons found in oil [30]. In situ exploitation of the bacteria, together with gross GBSs in field practice, might be highly promising in terms of oil-remediation efficiency [31,32].

In conclusion, due to the versatility of the GBSs in antimicrobial and emulsifying activities, the total GBS might be a promising alternative to chemical emulsifiers for application in various industries, including food processing and oil industries.

## 4. Experimental Section

### 4.1. Bacterial Isolation and Characterization

Soil and sediment samples were collected from mangrove forest, Qeshm Island, south of Iran (N 26°57′51″/E 56°27′to E 55°16′ of eastern longitude). The samples were dissolved in sterile seawater and then, bacterial strains were cultured on Muller Hinton agar made in seawater. Then, they were screened for antimicrobial activity using cross streak culture method [33] and an isolate with superior antimicrobial activity against *E. coli* was selected for further detailed examinations. Biochemical, morphological and 16S rRNA sequence analysis were conducted to identify the isolate. To amplify the 16S rRNA gene fragment, universal primers Fd1 (5′-AGAGTTTGATCCTGGCTCAG-3′) and Rd1 (5′-AGGAGGTGATCCAGCC-3′) were used and PCR process was programmed as described by Ebrahimipour et al. [9]. The PCR product was sequenced by Sinagene Company (Tehran, Iran) and nucleotide sequence was edited by BioEdit software version 5.0.6 (Abbott company, Calsbad, CA, USA). RNA sequence was analyzed using BLAST (http://blast.ncbi.nlm.nih.gov/Blast.cgi).

### 4.2. Media Preparation and Antimicrobial Activity

For preparation of antimicrobial compounds, a self-synthetic medium was designed based on Zobel marine broth containing (g·L^−1^) the following: glucose (10), peptone (5), yeast extract (5), K_2_HPO_4_ (1), MgSO_4_·7H_2_O (0.2), Na_2_CO_3_ (1) in sterile water with an initial pH of 7.0 ± 0.1 [34]. A loop of overnight inoculum was transferred into a 250 mL Erlenmeyer flask with 100 mL broth medium and incubated on a rotary shaker (100 rpm) at 35 °C for 72 h.

After centrifugation of the media at 5000 (rpm) for 15 min, the cell free supernatant was tested for antimicrobial activity against indicator pathogens such as *Escherichia coli* (ATCC25922), *Salmonella enteric* (ATCC13076), *Bacillus Subtilis* (ATCC465), *Bacillus cereus* (ATCC11778), *Candida albicans* (ATCC10231), *Pseudomonas aeruginosa* (ATCC85327) and *Staphylococcus aureus* (ATCC25923), *Aspergillus niger* (isolated from spoiled grape) using disc diffusion method. Additionally, the minimum inhibitory concentration (MIC) was determined [35].

### 4.3. Antimicrobial GBs Purification and Partial Characterization

The crude antimicrobial compound was decanted from the supernatant with equal volume of solvents such as ethyl acetate, chloroform and dichloromethane. The solvents were evaporated in a rotary vacuum evaporator and the dried powder was tested for detailed analysis. To further purify the sample, the resultant powder was passed through a silica gel column chromatography (1.5 × 30 cm; silica G-60; Merck, Darmstadt, Germany). The column was eluted with a mixture of chloroform/methanol/water by volume ratio of 65:25:5. Based on the disc diffusion results, the active fractions were taken and combined together. This process was repeated several times to reach maximum purity. These fractions were examined for the purity and preliminary determination of the GBSs, using thin layer chromatography (TLC). The developing phase for TLC was chloroform/methanol/water (70:10:2).

### 4.4. Instruments and Analytical Method

#### 4.4.1. Gas Chromatography-Mass Spectrometry (GC-MS)

The purified compound composition was determined through splitting it into lipophilic and hydrophilic (sugar) moieties followed by their related analysis by GC-MS. The lipophilic moiety was converted to fatty acid methyl esters (FAME) using anhydrous solutions of HCl/methanol (5% *w*/*v*) at 100 °C for 1 h in a boiling water bath [36]. FAME was extracted with n-hexane and the remaining phase was taken for derivatizing sugars into acetylated versions. The sugar moiety was converted to acetylated derivative [37]. FAMEs and sugar derivative were analyzed by a gas chromatograph (Agilent Technologies Inc., New York, NY, USA) equipped with a HP-5MS fused silica capillary column (60 cm × 0.25 mm ID × 0.25 film thickness, Agilent Technologies) with injector and detector temperatures of 280 °C and 300 °C, respectively, coupled to a mass spectrometer for mass scanning at a scan rate of 1.2 per second. The voltage of the detector was adjusted to 350 V. The oven temperature program was started from 130 °C and increased to 220 °C at a rate of 2 °C per minute. The carrier gas was nitrogen at a flow rate of 1 mL·min^−1^ and a split ratio of 50:1 [38].

#### 4.4.2. Fourier Transformed Infrared Spectroscopy (FTIR)

FTIR spectroscopy was carried out on a spectrometer (BRUKER, Karlsruhe, Germany) by KBr pellet method. The pellet was analyzed by measuring in the range of 4000–400 cm^−1^.

#### 4.4.3. Nuclear Magnetic Resonance Spectroscopy (NMR)

The bioactive compound was used for NMR spectroscopy by a BRUKER DRX-300 Avance spectrometer (BRUKER). The sample was dissolved in CDCl_3_ and Tetramethylsilane (TMS) was used as an internal standard. The ^1^H- and ^13^C-NMR spectrums were depicted at 400 and 150 MHz, respectively.

### 4.5. Physical Stability Studies of the Bioactive Compound

The purified glycolipid was examined for rheological properties such as surface tension reduction (STR) activity, emulsification activity (E24 index) and oil spreading diameter (OSD). STR activity was measured by a Fisher Scientific tensiometer (Fisher Scientific Co., Pittsburgh, PA, USA). E24 index and OSD were also determined as described by Ebrahimipour et al. [9]. GBS dissolved in distilled water (1 g·L^−1^) and was subjected to the following conditions: pH (4–12), temperatures (20–120 °C) and NaCl concentrations (0%–15%) for 1 h. The rheological properties of the treatment were then inspected using above-mentioned tests.

### 4.6. Optimization of GBS Production by Response Surface Method

Medium optimization for antimicrobial activity was carried out by a statistical design experiment, RSM. Five variables with significant effect, i.e., pH, temperature, peptone, NaCl and molasses concentration were selected to attain optimum condition for GBS production. The bacteria were grown in 250 mL flasks containing 100 mL broth medium shaken at 100 rpm and incubated for 72 h. As response, the emulsification activity related to each experimental run was determined followed by antimicrobial activity.

A half-milliliter of the cell free supernatant was taken, two-fold diluted serially and aliquots of 100 µL were added to sterile blank discs (6 mm). Upon drying, disc diffusion was performed against *E. coli* as reference strain. Then, the antimicrobial activity for each run was determined by plotting clear zone diameters against reciprocates of the dilution factors followed by calculating the area under the dilution curve (AUDC) as a response. The reason for employing such an approach is to avoid unknown external interferences related to difference in medium compositions resulted in varying viscosity of the media.

Subsequently, Design-expert 7.0 software was employed to find an appropriate model based on the central composite design (CCD). The experimental plan was drafted in 32 trials and all the experiments were conducted in triplicate. Their average was taken for calculating emulsification and antimicrobial activity value. The RSM responses were fitted by the following equation in terms of second-order polynomial equation:

$$Y=\beta_{0}+\underset{i}{\sum }\beta_{i}x_{i}+\underset{\mathrm{ii}}{\sum }\beta_{\mathrm{ii}}x_{i}^{2}+\underset{\mathrm{ij}}{\sum }\beta_{\mathrm{ij}}x_{i}x_{j}$$

where Y is the predicted response value, β_0_ is the intercept term, β_i_, β_ii_ and β_ij_ pertain to linear, quadratic and interaction coefficients for x_i_, x_i_^2^ and x_ij_ respectively.

### 4.7. Time-Course Study of GBS Production

The production of GBS was carried out in 250 mL Erlenmeyer flasks containing 100 mL of the optimal medium according to the RSM prediction with the following composition (g·L^−1^): Peptone (1%), yeast extract (0.5%), glucose (1%), NaCl (1%), K_2_HPO_4_ (0.1%), MgSO_4_·7H_2_O (0.02%) and Na_2_CO_3_ (1%). The best physical conditions for GBS production were achieved at pH 7, 33 °C and 100 rpm. Bioemulsification and antimicrobial activities as well as bacterial growth were measured during 72 h incubation at 8-h intervals.

## Figures and Tables

**Table 1:** Antagonistic activity of glycolipid BS produced by *Buttiauxella* sp. M44.

Test Organism	MIC (µg/mL)	Disc Diffusion (mm)
*E. coli*	200	18.5 ± 2.1
*S. enterica*	250	13.1 ± 3.3
*C. albicans*	150	23.5 ± 2.3
*A. niger*	100	26.2 ± 2.7
*B. Subtilis*	300	14.41 ± 3.2
*B. cereus*	250	10.60 ± 4.5
*S. aureus*	450	5.8 ± 1.7
*P. aeruginosa*	-	-

**Table 2:** Major fractions of GBS identified by GC-MS results.

Compound Name	Formula	Molecular Weight
Tetradecanoic acid methyl ester	C_15_H_30_O_2_	242
9-Hexadecenoic acid methyl ester	C_17_H_32_O_2_	268
Hexadecanoic acid methyl ester	C_17_H_34_O_2_	270
9-Octadecenoic acid methyl ester	C_19_H_36_O_2_	296
Octadecanoic acid methyl ester	C_19_H_38_O_2_	298
9,12-Octadecadienoic acid methyl ester	C_19_H_34_O_2_	294
d-Glucopyranose, 2, 3, 4, 5, 6 pentaacetate	C_16_H_22_O_11_	390

**Table 3:** ^1^H-NMR and ^13^C-NMR interpretation of GBS structure compared with those of d-glucose and octadecanoic acid.

Functional Group		d-Glucose		Glycolipid		Octadecanoic Acid	
		^1^H-NMR	^13^C-NMR	^1^H-NMR	^13^C-NMR	^1^H-NMR	^13^C-NMR
Sugar moiety	C-1, H-1	4.92	92.6	4.55	95.1	-	-
	C-2, H-2	3.53	73.5	3.13	72.8	-	-
	C-3, H-3	3.5	73.8	3.16	74.5	-	-
	C-4, H-4	3.15	70.8	3.13	69.8	-	-
	C-5, H-5	3.53	72.6	3.58	71.5	-	-
	C-6, (6-Hydroxyl)	1.29	61.6	**	65.2	-	-
Lipid moiety	1′ (Carboxyl)	-	-	**	172.8 *	11.00	180.5 *
	2′ (CH2)	-	-	2.26	34.2	2.35	35.4
	3′ (CH2)	-	-	1.56	26.3	1.64	24.7
	-CH2-(C′4-C′16)	-	-	1.25–1.27	29.0–33.1	1.26–1.32	33.9
	C′17 (CH2)	-	-	1.23	23.7	0.93	25.1
	C′18 (CH3)	-	-	0.86	14.0	0.88	15

* Meaningful chemical shift; ** remove of peaks related to glycosylic bond.

**Table 4:** Stability of GBS treated in different physical conditions.

**Properties**	**pH**			
	**4**	**7**	**8**	**12**
ST (mN/m)	18.4 ± 2.1	48.2 ± 3.8	50.5 ± 6.4	34.7 ± 4.0
OSD (mm)	10.1 ± 1.7	12.4 ± 1.2	12.6 ± 1.5	11.0 ± 2.2
E24 (%)	32.1 ± 3.3	46.4 ± 4.2	48.4 ± 5.4	22.7 ± 1.7
**Properties**	**Temperature (°C)**			
	**20**	**35**	**60**	**100**
ST (mN/m)	46.6 ± 4.8	53.5 ± 6.7	50.7 ± 6.5	10.0 ± 1.6
OSD (mm)	10.6 ± 2.5	9.7 ± 1.0	11.3 ± 2.3	12.5 ± 1.2
E24 (%)	48.3 ± 6.3	50.2 ± 7.1	54.8 ± 8.5	53.9 ± 6.6
**Properties**	**NaCl (%)**			
	**0**	**3**	**6**	**10**
ST (mN/m)	51.2 ± 7.6	48.4 ± 6.4	31.7 ± 5.2	28.6 ± 4.3
OSD (mm)	11.5 ± 1.7	12.2 ± 2.7	9.5 ± 0.8	8.6 ± 1.1
E24 (%)	44.7 ± 3.8	47.2 ± 6.5	33.5 ± 1.7	24.2 ± 3.0

**Table 5:** Experimental runs and results, actual and predicted, along with amounts of physical and nutritional variables.

Run	pH	Temperature (°C)	Peptone (g/L)	NaCl (%)	Molasses (%)	Actual Response	Predicted Response
1	7	35	0.5	3	1	11.34	11.08
2	6	30	0.75	4	1.5	15.06	15.75
3	6	30	1.25	2	1.5	12.67	13
4	6	20	0.75	2	1.5	4.42	3.59
5	6	30	0.75	2	1.5	14.49	13.49
6	5	25	1	1	1	10.4	10.78
7	8	30	0.75	2	1.5	16.39	16.45
8	6	30	0.75	2	1.5	12.17	13.49
9	5	35	1	3	1	8.28	7.89
10	6	40	0.75	2	1.5	0	1.02
11	6	30	0.75	0	1.5	17.07	16.57
12	7	35	1	3	2	10.84	10.26
13	5	25	1	3	2	8.47	8.34
14	7	35	0.5	1	2	16.95	16.78
15	6	30	0.75	2	1.5	14.22	13.49
16	6	30	0.75	2	0.5	15.33	15
17	6	30	0.75	2	1.5	14.05	13.49
18	7	25	0.5	3	2	15.81	15.81
19	5	25	0.5	1	2	8.38	8.66
20	6	30	0.75	2	1.5	13.78	13.49
21	7	35	1	1	1	16.24	16.16
22	5	35	0.5	1	1	8.21	8.24
23	5	35	1	1	2	7.06	6.76
24	6	30	0.25	2	1.5	13.4	13.25
25	5	35	0.5	3	2	6.25	5.78
26	4	30	0.75	2	1.5	6.81	6.92
27	7	25	1	1	2	12.54	12.72
28	7	25	0.5	1	1	9.13	9.64
29	6	30	0.75	2	1.5	12.41	13.49
30	6	30	0.75	2	2.5	13	13.51
31	5	25	0.5	3	1	12.38	12.58
32	7	25	1	3	1	14.6	14.69

**Table 6:** Statistical analysis (ANOVA) for evaluating the significance of variables.

**Source**	**Terms**	***p*-Value**	**Source**	**Terms**	***p*-Value**
Model	quadratic	<0.0001 *	X_2_X_4_	Interactive	<0.0001 *
pH (X_1_)	Linear	<0.0001 *	X_2_X_5_	Interactive	0.6633
Temperature (X_2_)	Linear	0.0057 *	X_3_X_4_	Interactive	0.0772
Peptone (X_3_)	Linear	0.7495	X_3_X_5_	Interactive	0.0008 *
NaCl (X_4_)	Linear	0.2946	X_4_X_5_	Interactive	0.1217
Molasses (X_5_)	Linear	0.0730	X_1_^2^	squared	0.0225 *
X_1_X_2_	Interactive	0.0044 *	X_2_^2^	Squared	<0.0001 *
X_1_X_3_	Interactive	0.5965	X_3_^2^	squared	0.6003
X_1_X_4_	Interactive	0.3503	X_4_^2^	Squared	0.0024 *
X_1_X_5_	Interactive	0.0030 *	X_5_^2^	squared	0.2817
X_2_X_3_	Interactive	0.8642	Lack of Fit	-	0.6260
**Parameter**	**Value**		**Parameter**		**Value**
Std. Dev.	0.46		R-Squared		0.9814
Mean	5.81		Adj R-Squared		0.9475
C.V. %	7.91		Pred R-Squared		0.7476
PRESS	31.51		Adeq Precision		21.158

* Denotes significant terms.
